# The “double jeopardy” of midlife and old age mortality trends in the United States

**DOI:** 10.1073/pnas.2308360120

**Published:** 2023-10-09

**Authors:** Leah R. Abrams, Mikko Myrskylä, Neil K. Mehta

**Affiliations:** ^a^Department of Community Health, Tufts University, Medford, MA 02155; ^b^Max Planck Institute for Demographic Research, Rostock 18057, Germany; ^c^Population Research Unit, University of Helsinki, Helsinki 00014, Finland; ^d^Max Planck – University of Helsinki Center for Social Inequalities in Population Health, Rostock 18057, Germany; ^e^Department of Epidemiology, University of Texas Medical Branch, Galveston, TX 77555

**Keywords:** life expectancy, mortality trends, aging population

## Abstract

Since 2010, US life expectancy growth has stagnated. Much research on US mortality has focused on working-age adults given adverse trends in drug overdose deaths, other external causes of death, and cardiometabolic deaths in midlife. We show that the adverse mortality trend at retirement ages (65+ y) has in fact been more consequential to the US life expectancy stagnation since 2010, as well as excess deaths and years of life lost in 2019, than adverse mortality trends at working ages. These results reveal that the United States is experiencing a “double jeopardy” that is driven by both mid-life and older-age mortality trends, but more so by older-age mortality. Understanding and addressing the causes behind the worsening mortality trend in older ages will be essential to returning to the pace of life expectancy improvements that the United States had experienced for decades.

Since 2010, US life expectancy has not increased appreciably, a sharp contrast to previous decades. Since 1900 and up until 2010, US life expectancy at birth steadily improved at an average rate of 2.8 y per decade (1, 2). Given the continuation of many favorable advances that have propelled life expectancy growth, including advances in medical care, the post-2010 stall in US life expectancy was unexpected by demographers. Much research has been focused on understanding the stall. In 2021, a National Academies of Science panel concluded that “[T]he stalling and subsequent decline in life expectancy during the 2010s appears to have been the product of an increase in mortality among middle-age and younger adults” (3). The report found that since 2012 there has been a widespread uptick in mortality at working age across geography, socioeconomic status, and race/ethnicity. The main causes of death contributing to the adverse trend at working ages have been drug overdose deaths, other deaths of despair (e.g., suicide, alcohol-related liver disease), and cardiometabolic diseases.

We show here that the United States has actually experienced an underappreciated “double jeopardy” in recent mortality, not only the well-studied adverse trend in mortality among working-age adults but also a significantly worsening trend in retirement ages (65+) (4). The relative contributions of changes in working and retirement-age mortality to the post-2010 US life expectancy stagnation have not previously been estimated. We show that, in fact, the adverse trend in retirement-age mortality has been more consequential to the post-2010 US life expectancy stagnation than the adverse trend in working-aged mortality. An improved understanding of the contribution of deaths at older ages to the US life expectancy stagnation shifts the explanatory framework of its causes, putting age-related chronic disease at the forefront.

## Results

Three population indicators of mortality are presented—life expectancy, excess deaths, and years of life lost (YLL)—to provide a comprehensive assessment of age-specific contributions under scenarios that weight age groups differently (5). The objective is to isolate the separate effects of trends in working and retirement-age death rates to the three indicators during 2010 to 2019 compared to 2000 to 2009, when life expectancy at age 25 (e_25_) increased by 1.36 y for women and 1.72 y for men.

**Fig. 1.**
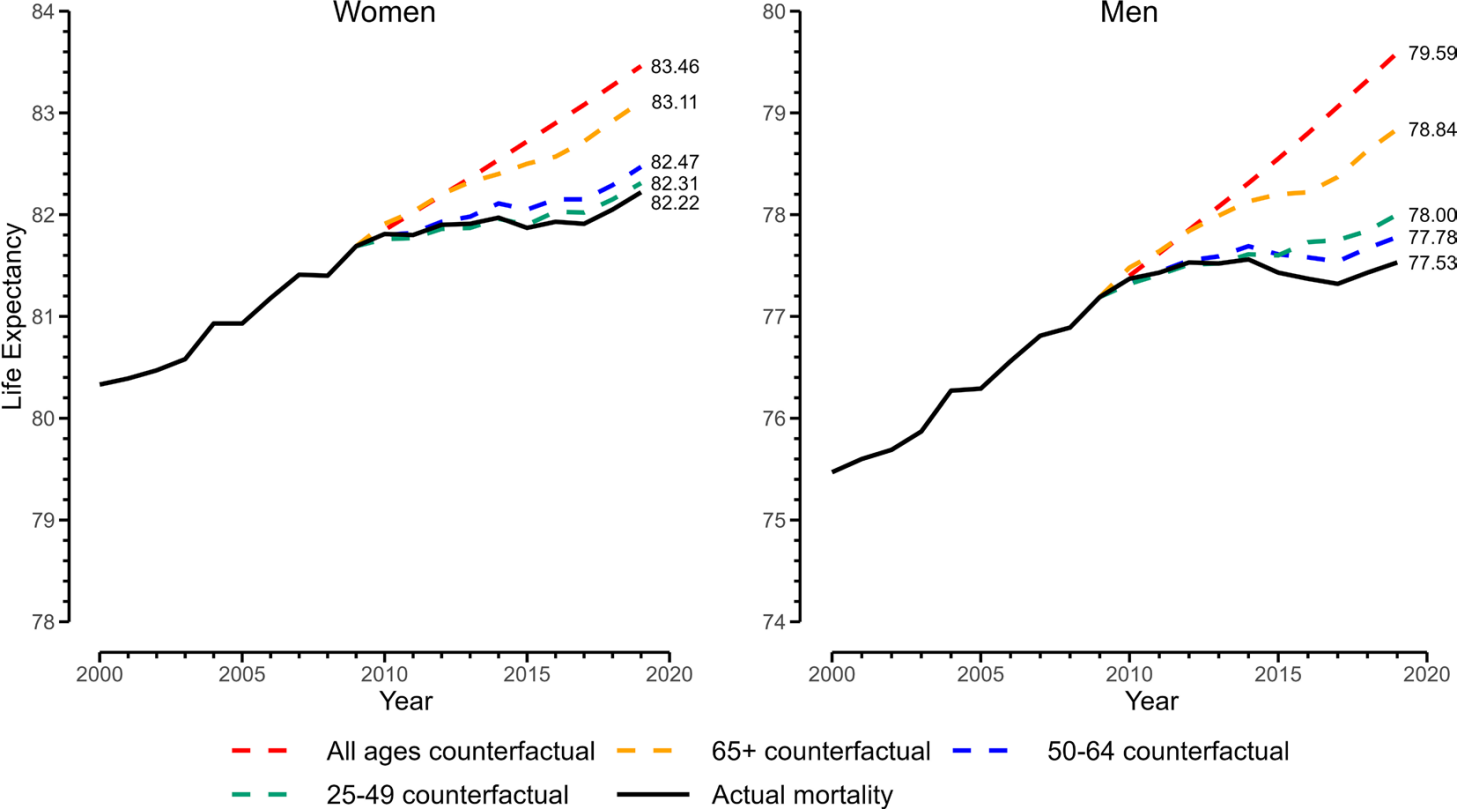
Real and counterfactual life expectancies at age 25, 2000 to 2019. Note: *y* axis is life expectancy at birth calculated from adding 25 y to e_25_ (life expectancy at age 25, conditional on surviving to age 25 y).

The Fig. 1 shows the results for e_25_. Between 2010 and 2019, e_25_ increased by 0.41 y for women and 0.17 y for men. The red dashed line indicates what e_25_ would have been if the 2000 to 2009 pace of age-specific mortality in ages 25+ (all observed ages) had continued into the 2010 to 2019 period. In this case, e_25_ in 2019 would have been 1.2 y higher for women and 2.1 y higher for men compared to the real values. Allowing only retirement-age (65+) mortality to continue at its 2000 to 2009 pace into 2010 to 2019, while holding working-age (25 to 64) trends at their real levels, isolates old age’s effect. In this scenario, depicted by the yellow dashed line (i.e., 65+ counterfactual), e_25_ in 2019 would have been 0.9 y higher than real life expectancy for women and 1.3 y higher for men, representing most of the total unrealized gain. If, on the other hand, working-aged mortality (25 to 49 and 50 to 64) were allowed to continue at its 2000 to 2009 pace and retirement mortality was left as observed, e_25_ in 2019 would have increased more modestly (blue and green dashed lines). Thus, while adverse trends at both the working and retirement ages have contributed to the overall e_25_ slowdown, the trend in retirement ages appears more consequential. In an additional analysis disaggregating ages 65 to 84 from 85+, we found that most of the effect for ages 65+ (75% for women and 90% for men) arose from the adverse trend in ages 65 to 84 as opposed to ages 85+ (6).

**Fig. 2.**
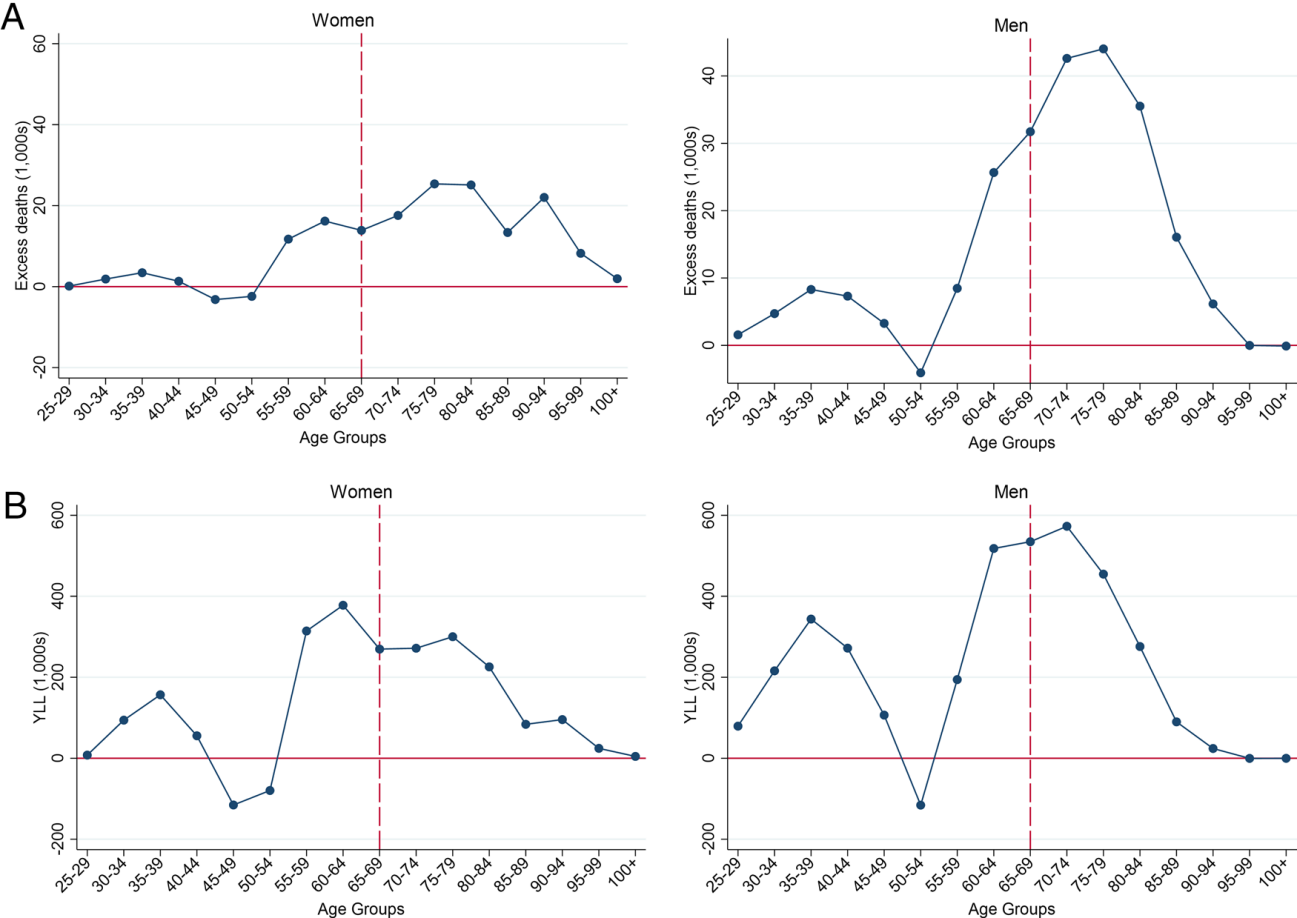
Excess deaths and YLL in 2019 due to adverse 2010 to 2019 mortality trends. Excess deaths (*A*). YLL (*B*). Note: Excess deaths were calculated using the real and counterfactual death rates in 2019 and actual population size in 2019, all by single year of age. YLL were calculated by multiplying the age-specific excess deaths with remaining life expectancy at each age from the actual 2019 period life table. Ages 65+ contributed 81% of excess deaths in women and 76% in men. Ages 65+ contributed 61% of YLL in women and 55% in men.

We next turn to excess deaths. Here, we compare real and counterfactual death rates’ impact on the actual 2019 population. Had age-specific mortality in ages 25+ continued their 2000 to 2009 pace after 2010, about 388,000 fewer deaths would have occurred in 2019. Fig. 2*A* breaks down the excess deaths by sex and age group, revealing that 81% of the nearly 157,000 excess deaths in women and 76% of the 230,000 excess deaths in men occurred at retirement ages (65+).

The Fig. 2*B* depicts YLL in 2019, the product of excess deaths and the difference between age at death and real life expectancy. Even though this calculation upweights deaths at younger ages, mortality in retirement ages still account for the majority of YLL, 61% of the about 2,100,000 y for women and 55% of the about 3,600,000 y for men.

## Discussion

Adverse trends at both working ages and retirement ages have held back progress in US mortality indicators, which we term a double jeopardy. Nonetheless, the effects of trends in retirement age outweigh those of working ages, even when metrics heavily weight loss of life at younger versus older ages. We found that, if retirement-age mortality had continued at its prior pace of decline, the United States would have experienced 0.9 y of increased life expectancy for women and 1.3 y for men between 2010 and 2019, rather than the observed stalled trend. Our counterfactual is by no means extreme. Countries such as Japan, Switzerland, Italy, and the United Kingdom have similar or higher life expectancy at age 65 than what our U.S. counterfactual produces.

The impact of old age mortality on the post-2010 US life expectancy stagnation is strong. It is also surprising because the United States has generally had advantages in old-age mortality compared to peer nations, making up for comparatively higher mortality at younger ages (7). By comparing the United States to itself at an earlier time point, we were able to contextualize the troubling pattern of older-aged US mortality and identify its disproportionately large effects on stagnating US life expectancy and related mortality metrics. While old age mortality is not explicitly rising in the 2010 to 2019 period, we observed a clear departure from earlier declines, with large implications. The COVID-19 pandemic, which disproportionately affected older Americans, likely compounded the preexisting mortality slowdown post-2019 (8).

Uncovering the impact of old age mortality has profound policy implications. Great research funding and policy efforts have been justifiably focused on turning the tides of the opioid epidemic and related deaths of despair. It is possible that some of the adverse trends observed in old age are associated with the same social and economic processes contributing to deaths of despair at working ages, such as wage stagnation, weak health care safety nets, declining social support from churches, civic organizations, and families (3). However, additional distal and proximal causes will be more relevant to older ages. Cardiovascular disease is an important driver of US life expectancy stagnation (9) and two-thirds of cardiovascular disease deaths occur after age 75, so risk factors such as cigarette smoking, physical inactivity, obesity, and diabetes may be central (10). In addition, old age mortality may be especially sensitive to changes in access to quality medical care and caregiving support. Future research could specifically compare causes of death and upstream factors contribution to adverse mortality trends in working versus older ages.

## Material and Methods

Annual mortality rates in the United States from 2000 to 2019 by sex and single-year age groups came from the Human Mortality Database (2). We calculated counterfactual death rates for 2010 to 2019 under a hypothetical that extrapolated the sex- and single-year-age-group-specific average annual change in all-cause mortality from 2000 to 2009 into 2010 to 2019. We applied these age-specific counterfactual mortality rates in a series of scenarios: all observed ages (25+), ages 25 to 49 only, ages 50 to 64 only, and ages 65+ only.

Life expectancies at age 25 conditional on surviving to age 25 in 2010 to 2019 were calculated using real mortality and the counterfactual mortality scenarios. Excess deaths were calculated at each single year of age as the difference between real deaths in 2019 and deaths that would have occurred by applying the 25+ counterfactual death rates to the actual population of 2019. YLL at each single year of age were calculated by multiplying excess deaths with remaining life expectancy in 2019 at that age. Excess deaths and YLL were summed within 5-y age groups in Fig. 2.

## Data Availability

Data used in this analysis are publicly available from the Human Mortality Database (2). Code and documentation are available at this link: https://osf.io/qpcrs/?view_only=311b623644e0438b​98b6c2333dd0915a.
